# Genome-scale metabolic network reconstruction of *Saccharopolyspora spinosa* for Spinosad Production improvement

**DOI:** 10.1186/1475-2859-13-41

**Published:** 2014-03-15

**Authors:** Xiaoyang Wang, Chuanbo Zhang, Meiling Wang, Wenyu Lu

**Affiliations:** 1 Department of Biological Engineering, School of Chemical Engineering and Technology, Tianjin University, Tianjin 300072, PR China; 2 Key Laboratory of system bioengineering (Tianjin University), Ministry of Education, Tianjin 300072, PR China; 3 Collaborative Innovation Center of Chemical Science and Engineering (Tianjin), Tianjin 300072, PR China

**Keywords:** *Saccharopolyspora spinosa*, Genome-scale metabolic network reconstruction, Target prediction, Strain engineering

## Abstract

**Background:**

Spinosad is a macrolide antibiotic produced by *Saccharopolyspora spinosa* with aerobic fermentation. However, the wild strain has a low productivity. In this article, a computational guided engineering approach was adopted in order to improve the yield of spinosad in *S. spinosa*.

**Results:**

Firstly, a genome-scale metabolic network reconstruction (GSMR) for *S.spinosa* based on its genome information, literature data and experimental data was extablished. The model was consists of 1,577 reactions, 1,726 metabolites, and 733 enzymes after manually refined. Then, amino acids supplying experiments were performed in order to test the capabilities of the model, and the results showed a high consistency. Subsequently, transhydrogenase (PntAB, EC 1.6.1.2) was chosen as the potential target for spinosad yield improvement based on the *in silico* metabolic network models. Furthermore, the target gene was manipulated in the parent strain in order to validate the model predictions. At last, shake flask fermentation was carried out which led to spinosad production of 75.32 mg/L, 86.5% higher than the parent strain (40.39 mg/L).

**Conclusions:**

Results confirmed the model had a high potential in engineering *S. spinosa* for spinosad production. It is the first GSMM for *S.spinosa*, it has significance for a better understanding of the comprehensive metabolism and guiding strain designing of *Saccharopolyspora spinosa* in the future.

## Background

Spinosad is a macrolide antibiotic produced by *Saccharopolyspora spinosa* with aerobic fermentation. Due to its unique chemical structure and mechanism, it can control diamondback moth; beet armyworm and other lepidopteron pests effectively, while have non-toxic to mammals and birds [1]. Therefore spinosad has been considered to be the most effective biological pesticides after avermectin, becoming a new hot issue in the research and development of biological pesticides. The compounds got the "Presidential Green Chemistry Challenge Award" of the United States in 1999, because it has the chemical pesticide’s fast-acting and the biopesticide’s safety, low residual properties. So its derivative got the award again in 2008 [2].

Spinosyns A and D, the two major components in the *S. spinosa* fermentation,are defined as spinosad [3]. It mainly contains a 21-carbon tetracyclic with two deoxysugars: tri-O-methylated rhamnose and forosamine [4]. Waldron firstly analyzed the proposed spinosyn biosynthetic pathway [5], In recent years, the spinosad biosynthetic pathway has been clarified more accuracy: *SpnA*, spnB, spnC, spnD, and spnE responsible for type I polyketide synthase; spnF,spnJ, spnL, and spnM for modifying the polyketide synthase product [6]; spnG, spnH, spnI, and spnK for rhamnose attachment and methylation [7]; spnP, spnO, spnN, spnQ, spnR, and spnS for forosamine biosynthesis; gtt, gdh, epi, and kre for rhamnose biosynthesis [8] and beside the spinosad gene cluster four genes ORF-L16, ORF-R1, and ORF-R2, have no effect on spinosad biosynthesis.

The wild stain of *Saccharopolyspora spinosa* has low spinosad productivity, so lots of work has been done in strain modification. Three strategies have been mainly used in the spinosad improvement: (a) Traditional physical and chemical mutagenesis [9]; (b) Optimization of fermentation process [10,11]; (c) Improving the spinosad production by the genetic engineering method. Pan [12] got a over threefold spinosad production by introducing the additional *gtt* and *gdh* genes under the control of PermE* promoter. Tang [13] increased the spinosyn production by 3.8-fold through overexpression of the spinosyn biosynthetic genes participating in the conversion of the cyclized polyketide to spinosyn, obtained by direct cloning via Red/ET recombination. Xue [14] duplicated sp*nP, spnQ, spnN, spnQ, spnR, spnS, spnK, gtt, gdh* and *kre* genes under the control of PermE* promoter, spinosad production was a 5.0-fold enhancement compared with the wild-type S. spinosa. However, the spinosad is a secondary metabolite which has complex metabolic pathways. It is difficult to satisfy the industrial demands if we only pay attention to a few gene modifications.

In recent years, more and more whole genomes have been sequenced with the development of high-throughput sequencing technology and reduction of sequencing prices. It is a hot topic to study how to use such a large genome database. The genome-scale metabolic network (GSM) constructed based on genome annotation information provide an integral level to review organism metabolism. Now Genome-Scale-Metabolic-Model (GSMM) reconstruction technology has a great progress no matter in speed but quality [15,16]. At present, many articles has elaborated GSMM establishment process [17,18], these provided foundation to build a precision standardized GSMM. GSMM is widely used to aid strain transformation in metabolic engineering as a systems biology tool [19,20]. Brochado [21]used GSMM successfully predict several genes targets in Bread yeast, which increased the vanillin yield about 5-fold. Huang used the GSMM of *Streptomyces tsukubaensis* to improve the FK506 production which led to a 1.47 fold increase [22]. The similar way was also used to increase the expression of the green fluorescent protein in *lactococcus lactis*[23]. GSMM reconstruction is a time consuming work, the metabolic reconstruction for secondary metabolite producing still requires extensive manual refinement, because the lack of genome annotation information.

There were no reports on the refined GSMM for *Saccharopolyspora spinosa*. In this work, the first manually refined GSMM for *Saccharopolyspora spinosa* was established. The amino acid supplementation experiments were conducted to verify the module. Then a potential target gene transhydrogenase was assessed. Overexpression of this gene led to 86.5% higher spinosad yield than that of the wild stain.

## Results and discussion

### Character of the GSMM

**Figure 1:**
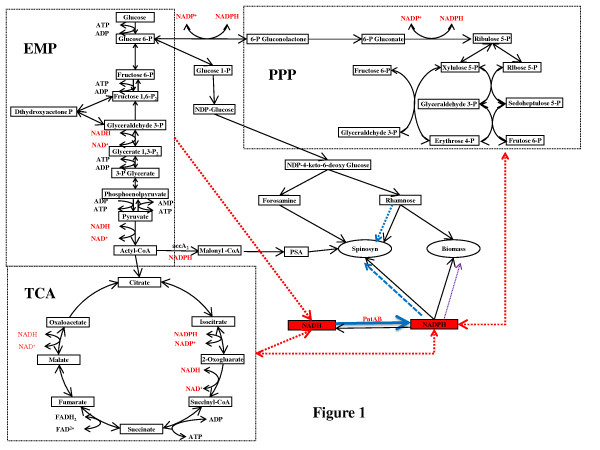
**Schematic diagram of metabolic pathways for S.spinosa.** The NADH/NADPH generation and consumption are showed in red. The enhanced pathway is showed in blue (solid line represents gene overexpression, dotted line means possible enhanced pathways). The weakened pathway is showed in purple.

The first GSMM for *Saccharopolyspora spinosa* using a highthroughput protocol was constructed. The GSMM contained 1577 metabolites and 1736 reactions and 733 enzymes (Additional file 1 shows the network in more detail). Given that the spinosad was produced alongside biomass, 42 reactions were required to fill the model gaps. Major metabolic pathways for *S.spinosa*, including the glycolytic pathway, pentose phosphate pathway (PPP), tricarboxylic acid (TCA) cycle, and spinosad biosynthesis, are illustrated in Figure 1. Additionally five subsystems were divided, containing: gluconeogenesis, amino acid synthesis pathway, fatty acid synthesis pathway, phospholipid synthesis pathways and nucleotide synthesis pathways. The Character was summarized in Table 1.

**Table 1:** **Network summary for *S.spinosa *** **GSMM**

**Metabolic network characteristics**	**Total**
Metabolites	1737
Reactions	1577
Intracellular reactions	1377 (87.3%)
Transport reactions	105 (6.6%)
Exchange reactions	95 (6.1%)
Reactions added filled by manual curation	42
Enzymes	733

### Amino acid addition *in silico* simulation and experiments

**Figure 2:**
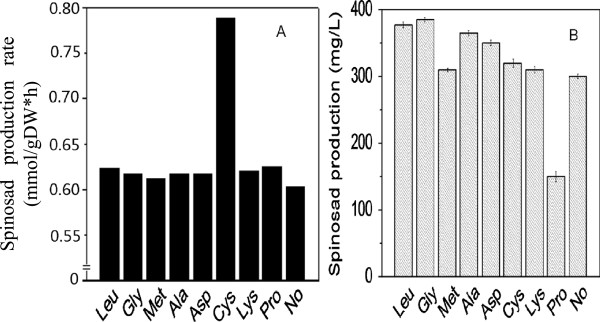
**Amino acids supplement experiment. A**: *In silico* simulations of the effect on spinosad biosynthesis after supplementing individual amino acid. **B**: Spinosad production after supplementing each individual amino acid.

To validate the spinosad biosynthetic pathway, we used the GSMR model to predict the potential benefit of supplementing the media with individual amino acid (AA) in terms of spinosad production. Eight AAs (Leu, Gly, Met, Ala, Asp, Cys, Lys, Pro) were individually added to study the effect on spinosad production. The experiments and *in silico* simulation results were showed respectively in Figures 2B and A. The experiment dates showed that Gly was the best amino acid to spinosad production and following Leu, Ala. Pro had a negative effect on spinosad production. The remaining four amino acids (Asp, Met, Cys, Lys) displayed no significant effects on spinosad production.

Amino acids play an important role in antibiotic synthesis and cell growth in the fermentation process as important biological small molecules. AA can directly participate in the biosynthesis of antibiotics as the precursor [24,25]; AA or its peptide structure influence the biosynthesis of antibiotics by regulating the activity of the enzymes in the pathway as activators or inhibitors and its hydrolysates can change microbial growth environment by altering the PH in the medium. The main product in glycine metabolism is acetyl-COA which is the precursor of spinosad [26], this may explain the results in our work. Hinnebusch [27] proved that the *GCN4* gene which encodes a positive regulator of unlinked amino acid biosynthetic genes in yeast is itself regulated by amino acid availability and that the regulation occurs at the translational level. Proline inhibit the spinosad synthesis may due to it or its metabolites participate in the regulation process. The GSMM in our work has no regulation systems, so the result was not consistent with the experiments.

### Impact of PntAB gene on spinosad synthesis

**Figure 3:**
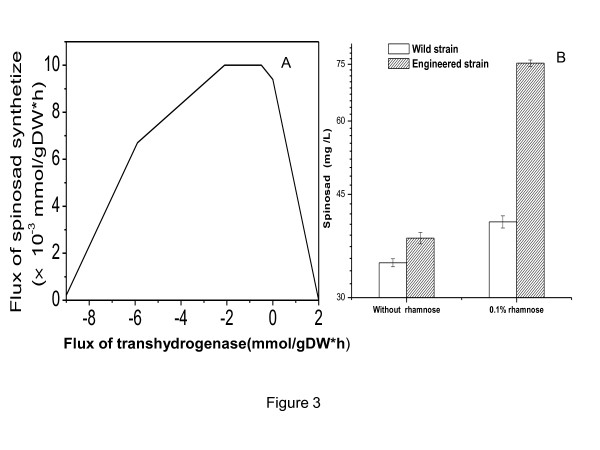
**The effect of reducing power level on spinosad synthesis. A**: Impact of transhydrogenase activity on spinosad synthesis (*in silico*) **B**: The experimental results of shake flask fermentation.

Spinosad is a secondary metabolite. Energy and reducing power NADPH were required in its synthesis process. Cellular reducing power level, such as NADPH/NADP^+^ and NADH/NAD^+^, has an important role in the metabolic regulation in the cell. Therefore the reaction R00112 (NADPH + NAD^+^ = NADP^+^ + NADH) was used as control variables, the spinosad synthesis reaction as a target reaction to study the effect of transhydrogenase activity on spinosad synthesis. The results were exhibited in Figure 3A.

In the Figure 2A, when the reaction R00112 flux is 0, the spinosad synthesis rate didn’t reach the peak. When the reaction flux is positive (NADPH → NADH), the spinosad synthesis rate decreased. In contrast, the spinosad synthesis rate climb up and then decline when the the reaction flux is negative. Further conclude can be obtained, spinosad was produced in the stationary phase, at this phase, transhydrogenase in the cell kept the balance of the reducing power(NADPH/NADH), but the balance point was not the best for spinosad synthesis. In order to prove the simulation results, *pntAB* was over expressed, the yield of spinosad in *S.spinosa* 261P increased to 37.9 mg/L which was little higher than the wild strain(34.4 mg/L). Then 0.1% rhamnose was added, the yield increased to 75.32 mg/L which was 0.86-fold higher than that in the wild-type S. spinosa (40.39 mg/L).

Efficient regeneration of NADPH which mainly comes from PPP and TCA pathways plays an important role in biotransformation processes. The biotransformation is often limited by cofactor regeneration because cells would rather grow better than intend to sustain the biotransformation [28,29]. The NADPH regeneration system needs to be enhanced in order to satisfy biotransformation. Three ways were modified to increase the NADPH level in the cell including oxidative part of the pentose phosphate pathway (PPP) [30,31], the tricarboxylic acid cycle (TCA) [32], and the transhydrogenases system. Overexpression of *pntAB* in *E. coli* got a 3.5-fold conversion of acetophenone to (R)-phenylethanol [33]. The similar way was conducted in *Corynebacterium glutamicum* to get a high yield of L-lysine [34]. Bastian [35] obtained a 6.5-fold enhancement of isobutanol production under anaerobic conditions in E. coli by overexpression of *pntAB*. In our work, two reasons may explain the results that the engineered strain has a low production in general fermentation medium: Firstly, spinosad is a secondary metabolite and it has a low synthesis rate; Secondly, NADPH was mainly used in biomass synthesis.

Rhamnose is both the precursor of spinosad and the cell wall ingredient, but the *Saccharopolyspora spinosa* only have one set of rhamonose gene [8,36]. The yield of spinosad had a 3.5-fold enhancement by duplication of the rhamnose biosynthetic genes (*gtt* and *gdh*) in the previous study [14]. So the rhamnose plays an important role in spinosad synthesis. The rhamnose in the fermentation medium can accelerate the use of pseudoaglycones (PSA). Then PSA synthesis made more NADPH flow into spinosad synthesis. Reducing power balance changed by over expressing *pntAB*, more NADPH was produced from NADH. When the rhamnose was added, more NADPH flowed into spinosad synthesis pathway, so the engineered strain had a high productivity in rhamnose medium.

## Conclusions

In this work, the first Genome-scale metabolic network of *Saccharopolyspora spinosa* was constructed following a systematic workflow. Then it was validated by amino acids supplementation experiments. Finally based on the GSMM, *PntAB* was identified as the target gene. The strain was engineered according to the predicted target. Fermentation characterization of the engineered strain showed the improved capacities of spinosad production. The final yield of spinosad is 75.32 mg/L which is increased by 86.5% than the parental strain. Our results show the genome-scale metabolic network model is a powerful tool to guide strain engineering towards improved bio-production in *S.spinosa*, as well as other microorganisms.

## Materials and methods

### Genome-scale metabolic network reconstruction

The genome of *Saccharopolyspora spinosa* was sequenced and annotated in 2011 by Yuanlong Pan [37], the results can be got in NCBI [38]. The complete sequence of the 8.809 Mb *Saccharopolyspora spinosa* NRRL 18395 genome has a 67.94% GC content. The model reconstruction followed the standard protocols described previously [17]. The process of the genome-scale metabolic network reconstruction for the *Saccharopolyspora spinosa* can be described as follows:

Firstly, a draft reconstruction was created. The necessary information, containing genome annotation information and the biochemistry information of the enzymes, can be searched in KEGG and NCBI. The following factors were included in the draft: (1) the basic reaction information (ID, name, equation and reversibility), (2) the enzymes and genes relevant to particular reaction. (3) the subsystems. All the information above was saved in an Excel which contains 1830 reactions and 1849 metabolites.

Secondly, refine the draft model got above. Many reactions were redundant in the draft while some necessary ones were absent, so a lot of work was done in the refine process. The reactions and metabolites IDs may different in different database, only one ID was remained; The reactions which synthesize or modify the macromolecules were replaced by the biomass formulation reactions; Only one form of the multi-step reaction was remained in the model. For example, the overall reaction R004385 was kept while its sub-step reactions R00174 were removed; At last, reactions’ reversibility and cofactors were determined and verified.

At the last stage, spontaneous reactions, exchange reactions, transport reactions, biomass reactions and spinosad synthesis reactions were added to get a complete GSMM. Biomass formation was mainly based on the chemicals of the cell. The reactions containing the metabolites which were verified to be taken up from the medium and the metabolite which can diffuse through the membranes were added as the transport reaction. Exchange reactions were added for all extracellular metabolites, which represented the system boundaries.

### Biomass equation

The biomass equation information was mainly got from available literature date. It is divided into proteins, RNA, DNA, lipid cell wall peptidoglycanpolysaccharide muramic acid. DNA and RNA composition were determined based on genomic data from the *Saccharopolyspora spinosa* genome. Protein composition was determined based on open reading frames (ORFs). Other components were adapted from *S. coelicolor*[39]. The biomass composition is described in detail in the Additional file 2.

### Constraint-Based Flux Analysis

Constraint-Based Flux Analysis (FBA) has a wildly use in flux distribution calculations [40,41]. The resulting model was analyzed using Constraint-Based Reconstruction and Analysis (COBRA). Robustness prediction was performed using the COBRAToolbox-2.0 in MATLAB, with GLPK and CPLEX as the optimization programming solvers [42]. The stoichiometric matrix (S) and reaction flux constrains were extracted from the SBML file. At the model curation process, FBA was used to fill the gaps until the model can use the nature medium to synthesize biomass and spinosad. Robustness [43] analysis is an algorithm based on FBA, the flux through a selected reaction is varied and the optimal objective value is calculated as a function of this flux. This method can be used to simulate the influence of the biological metabolic state caused by internal or external disturbance.

### Bacterial strains, plasmids, media and culture conditions

*Escherichia coli* DH5α was used for all plasmid constructions and amplification. *E. coli* ET-12567 was used as the door strain in biparental intergeneric conjugations. *S. spinosa* ATCC49460 was used as the parent strain. The pOJ261, which is pOJ260 with ermE* promoter, was used as expression plasmid. The ermE* promoter was digested from pIB139. Plasmids and stains used in this study are summarized in Table 2.

**Table 2:** The strains and plasmid used in this study

**Strain or plasmids**	**Description**	**Source or reference**
**Strains**		
*E. coli* DH5*α*	Host for general cloning	TransGen biotech
*E. coli* ET-12567	Donor stain for conjugation between *E. coli* and *S. spinosa*	[44]
*S.spinosa* ATCC 49460	Wild strain	ATCC
*S.spinosa* 261P	*S.spinosa* ATCC 49460 harboring pOJ261P	This study
**Plasmid**		
pOJ260	*E. coli*–Streptomcyes shuttle vector; apr oriT repPUC *lacZ*	[45]
pOJ261	pOJ260 with ermE* promoter	Our lab
pOJ261P	pOJ260 with ermE*-controlled *PntAB*	This study

*E. coli* strains were cultured in Luria-Bertani (LB) medium at 37°C. The slant and seed medium contained (g/L): trypticase soy broth, 30; yeast extract, 3; MgSO^4^ · 7H^2^O, 2; glucose, 10; and maltose, 4, pH 7.2. In the slant medium 20 g/L agar was added. The fermentation medium contained (g/L): glucose, 68; cottonseed flour, 22; peptone C, 25; corn seed liquor, 14.5; methyloleate, 40; and CaCO3, 5, pH 7.2. ABB13 medium contained (g/L): soy peptone, 5; soluble starch; CaCO_3_, 3; MOPS, 2.1; agar 20.

Strains were cultured on a 220 rpm rotary shaker at 29°C in a 250 mL flask containing 30 mL of seed medium for 3 days. Then 3 mL of seed medium was injected into 30 mL fermentation media in a 250 mL flask. Strains were cultured for 9 days at 29°C on a 220 rpm rotary shaker. The fermentations were run in triplicate [14].

### Amino acids addition

After 72 h fermentation, 0.05% of amino acids (Leu, Gly, Met, Ala, Asp, Cys, Lys, Pro) Standards was added by 0.22 μ filter membrane filtration.

### Gene cloning, plasmid construction and transformation

General DNA manipulation was performed according to the standard protocols [46]. PntAB was amplified from genomic DNA of *S. spinosa* using primer pairs of F-CCTA*TCTAGA*CGCCGGACAAGGACGl-XbaI,R-GGATC*CATATG*ACCTCTCCGGAGAGC-NdeI(The italics parts represent the restriction enzyme cutting site). The PCR product of *PntAB* was digested by Ndel and Xbal. Then, it was cloned into pOJ261 which was also digested by Ndel and Xbal to get pOJ261P.

The constructed plasmid was introduced into S. spinosa strains by conjugation from E. coli ET12567 and homologous recombination into the chromosome as described as [44].The recombination stain *S.spinosa 261P* was confirmed by PCR amplification with vector primers V-F:CCGTGATTTTGTAGCCCTGG and V-R: GGCCTACTTCA CCTATCCTGC, and the result was positive.

### Analysis of spinosad biosynthesis in fermentation cultures

Spinosad in the fermentation broth was determined as described [10]. Every level in the experiment was repeated three times, so the figures in the article were the mean value of the experiment, Standard deviations were calculated from triplicate flasks.

## Competing interests

The authors declare that they have no competing interests.

## Authors' contributions

WYL and XYW conceived and designed the research. XYW and CBZ performed the model construction and calculation. Amino acids supplement experiments and strain engineering was conducted by MLW and XYW respectively. CBZ analyzed the date and drafted the manuscript. WYL supervised the research and revised the manuscript. All authors read and approved the final manuscript.

## Supplementary Material

Additional file 1 The SPNV1.0 model content. Click here for file

Additional file 2 **Biomass composition of *Saccharopolyspora spinosa.***
Click here for file
