# Supplementary material for: Genome-scale metabolic network reconstruction of Saccharopolyspora spinosa for Spinosad Production improvement
Source: Microb Cell Fact. 2014 Mar 15;13:41. doi: 10.1186/1475-2859-13-41 (PMC4003821; doi:10.1186/1475-2859-13-41)
Supplement: Additional file 2 — Biomass composition of Saccharopolyspora spinosa. [file 1475-2859-13-41-S2.doc]

**Biomass composition**

A complete cellular composition of a *Saccharopolyspora spinosa* could not be found in the literature. Therefore the content of macromolecular biomass components was taken from different sources (Borodina et al. 2005).

| **Component** | **g·g-1 DW** |
| --- | --- |
| **Protein** | **0.425** |
| **DNA** | **0.037** |
| **RNA** | **0.172** |
| **Lipids** | **0.047** |
| **Polar (Phospholipids)** | **0.028** |
| **Non-polar (TAGs)** | **0.019** |
| **Cell wall** | **0.226** |
| **Peptidoglycan** | **0.113** |
| **Carbohydrates** | **0.045** |
| **Teichoich acid** | **0.068** |
| **Ash** | **0.009** |
| **SUM** | **1.000** |

**Biomass equation**

0.425 Protein + 0.172 RNA + 0.037 DNA + 0.028 Phospholipid + 0.019 Triacylglycerol + 0.113 Peptidoglycan + 0.045 Polysaccharide + 0.068 Teichoic acid + 47 ATP = Cell + 47 ADP + 47 Orthophosphate

**DNA composition**

The DNA composition was calculated based on the genome information of *S.spinosa*. The polymerisation energy was assumed to be the same as in *E. coli* (Ingraham et al. 1983).

| **Nucleotide** | **mol/mol DNA** | **MW, g/mol** | **mol/g DNA** |
| --- | --- | --- | --- |
| **dAMP** | **0.160** | **313.2** | **0.519** |
| **dCMP** | **0.340** | **289.2** | **1.099** |
| **dTMP** | **0.160** | **304.2** | **0.519** |
| **dGMP** | **0.340** | **329.2** | **1.099** |
| **Energy requirement for polymerization(ATP): 4.40** | | | |

**DNA biosynthesis equation**

0.519 dATP + 1.099 dCTP + 0.519 dTTP + 1.099 dGTP + 4.4 ATP = 4.4 ADP + 4.4 Orthophosphate + 3.236 Diphosphate + DNA

**Protein composition**

The amino acid composition was determined from translated proteins in the *Saccharopolyspora spinosa* genome. The polymerisation energy was assumed to be the same as in *E. coli* (Ingraham et al. 1983).

| **Amino acid** | **%protein (w/w)** | **MWa, g/mol** | **Translated proteins** | **mmol/g proteins** |
| --- | --- | --- | --- | --- |
| **Alanine** | **13.19** | **71.09** | **357920** | **1.230** |
| **Arginine** | **8.35** | **156.20** | **226932** | **0.778** |
| **Asparagine** | **1.78** | **114.12** | **48416** | **0.166** |
| **Aspartate** | **5.99** | **115.10** | **162307** | **0.558** |
| **Cysteine** | **0.81** | **103.16** | **22294** | **0.076** |
| **Glutamate** | **2.84** | **128.15** | **77240** | **0.264** |
| **Glutamine** | **6.03** | **129.13** | **163747** | **0.562** |
| **Glycine** | **9.46** | **57.07** | **256735** | **0.882** |
| **Histidine** | **2.32** | **137.16** | **63313** | **0.217** |
| **Isoleucine** | **3.19** | **113.18** | **86763** | **0.297** |
| **Leucine** | **10.48** | **113.18** | **285107** | **0.977** |
| **Lysine** | **1.72** | **128.19** | **47014** | **0.160** |
| **Methionine** | **1.68** | **131.21** | **45535** | **0.157** |
| **Phenylalanine** | **2.91** | **147.19** | **79208** | **0.271** |
| **Proline** | **5.88** | **97.13** | **159918** | **0.549** |
| **Serine** | **5.24** | **87.09** | **142734** | **0.489** |
| **Threonine** | **5.51** | **101.12** | **150088** | **0.514** |
| **Tryptophan** | **1.52** | **186.23** | **41426** | **0.142** |
| **Tyrosine** | **1.90** | **163.19** | **51772** | **0.177** |
| **Valine** | **9.20** | **99.15** | **249998** | **0.858** |
| **Energy requirement for polymerization(ATP): 40** | | | | |

**Protein biosynthesis equation**

1.230 L-Alanine + 0.778 L-Arginine + 0.166 L-Asparagine + 0.558 L-Aspartate + 0.076 L-Cysteine + 0.264 L-Glutamate + 0.562 L-Glutamine + 0.882 Glycine + 0.217 L-Histidine + 0.297 L-Isoleucine + 0.977 L-Leucine + 0.160 L-Lysine + 0.157 L-Methionine + 0.271 L-Phenylalanine + 0.549 L-Proline + 0.489 L-Serine + 0.514 L-Threonine + 0.142 L-Tryptophan + 0.177 L-Tyrosine + 0.858 L-Valine + 40.0 ATP + 49.3 H2O = 40.0 ADP + 40.0 Orthophosphate + Protein

**RNA composition**

The RNA composition was determined from genomic data assuming the following composition: 5% mRNA, 75% rRNA and 20% tRNA. The nucleotide composition of mRNA was taken as for genomic DNA. The nucleotide composition of rRNA was calculated from the sequences of 16S, 23S and 5S ribosomal RNA units. tRNA composition was found from sequences of leucine and glycine transporting RNAs. The polymerisation energy was assumed to be the same as in *E. coli* (Ingraham et al. 1983).

| **Nucleotide** | **mol/mol RNA** | | | **MWa, g/mol** | **mol/mol RNA** | **mmol/g RNA** |
| --- | --- | --- | --- | --- | --- | --- |
|  | **mRNA** | **rRNA** | **tRNA** |  |  |  |
|  | **5%** | **75%** | **20%** |  |  |  |
| **AMP** | **0.160** | **0.212** | **0.171** | **329.2** | **0.201** | **0.702** |
| **GMP** | **0.340** | **0.210** | **0.354** | **345.2** | **0.245** | **0.854** |
| **CMP** | **0.340** | **0.224** | **0.285** | **305.2** | **0.242** | **0.844** |
| **UMP** | **0.160** | **0.210** | **0.190** | **306.2** | **0.203** | **0.708** |
| **Energy requirement for polymerisation (ATP):** | | | | | | **1.25** |

**RNA biosynthesis equation**

1.952 ATP + 0.854 GTP + 0.844 CTP + 0.708 UTP = 1.25 ADP + 1.25 Orthophosphate + 3.108 Diphosphate + RNA

**Phospholipid composition**

Phospholipid composition was modified from *S. colelicolor* (Borodina et al. 2005)

| **Component** | **g/g phospholipids** | **mmol/g phospholipids** |
| --- | --- | --- |
| **Phosphatidylethanolamine** | **0.75** | **1.089** |
| **Phosphatidylglycerol** | **0.18** | **0.250** |
| **Cardiolipin** | **0.07** | **0.052** |

Phospholipid biosynthesis equation

1.089Phosphatidylethanolamine +0.052 Cardiolipin + 0.250 Phosphatidylglycerol = Phospholipids

**Triacylglycerols (TAGs) composition:**

TAGs composition was obtained from *S. colelicolor.* (Borodina et al. 2005)

| **TAG components** | **mol/mol TAG** | **mmol/g TAG** |
| --- | --- | --- |
| **Glycerol-3-phosphate** | **1** | **1.244** |
| **C14** | **0.040** | **0.050** |
| **C15** | **1.349** | **1.677** |
| **C16** | **0.338** | **0.421** |
| **C17** | **1.262** | **1.570** |
| **C18:1** | **0.011** | **0.014** |
| **Average molecular weight:** | **803.9** |  |

**TAG** **biosynthesis equation**

1.244sn-Glycerol 3-phosphate + 0.050Tetradecanoyl-[acp] + 1.677C150ACP + 0.421 Hexadecanoyl-[acp] + 1.570C170ACP + 0.014Oleoyl-[acyl-carrier protein] = Triacylglycerol + 3.732 Acyl-carrier protein + 1.244 Orthophosphate

**Cell wall composition**

The cell wall composition was mainly obtained from *S. colelicolor* (Borodina et al. 2005). The energy of polymerization form *E. coli* (Ingraham et al. 1983)

**Peptidoglycan composition:**

| **Component** | **Molar ratio in peptidoglycan of S. colelicolor** | **MWa, g/mol** | | **mmol/g peptidoglycan** |
| --- | --- | --- | --- | --- |
| **N-acetylmuramic acid** | **0.9** | | **275** | **1.007** |
| **N-acetylglucosamine** | **1.1** | | **203** | **1.197** |
| **Alanine** | **1.7** | | **71** | **1.900** |
| **Diaminopimelinic acid** | **1.0** | | **154** | **1.140** |
| **D-glutamate** | **0.9** | | **129** | **1.014** |
| **Glycinea** | **0.9** | | **39** | **0.973** |
| **Energy requirement for polymerisation (ATP): 5.026** | | | | |

**Peptidoglycan biosynthesis equation:**

1.007UDP-N-acetylmuramate + 1.197 UDP-N-acetyl-D-glucosamine + 1.900 D-Alanyl-D-alanine + 0.950 L-Alanine + 1.140 meso-2,6-Diaminoheptanedioate + 1.014 D-Glutamate + 0.973 Glycine + 5.026 ATP = Peptidoglycan + 0.950 D-Alanine_EXE + 1.197 UDP + 1.007 UMP + 5.026 ADP + 5.026 Orthophosphate

**Carbohydrates composition:**

| **Component** | **Molar ratio** | **MWa, g/mol** | **mmol/g carbohydrate** |
| --- | --- | --- | --- |
| **N-acetylglucosamine** | **1** | **203** | **1.897** |
| **Galactose** | **2** | **162** | **3.794** |

**Carbohydrates in cell wall equation:**

1.897 UDP-N-acetyl-D-glucosamine + 3.794 UDP-D-galactose = 5.691 UDP + Polysaccharide

**Teichoic acid composition:**

| **Components** | **molar ratio** | **MWa, g/mol** | **mmol/g teichoic acid** |
| --- | --- | --- | --- |
| **Polyglycerophosphate chain** | **1** | **1849** | **0.518** |
| **Lysine** | **0.25** | **128** | **0.129** |
| **N-acetylglucosamine** | **0.25** | **203** | **0.129** |
| **Energy requirement for polymerisation (ATP):** | | | **0.129** |

**Teichoic acid biosynthesis equation:**

6.216sn-Glycerol 3-phosphate+6.216CTP + 0.129L-Lysine + 0.129 UDP-N-acetyl-D-glucosamine + 0.129 ATP = Teichoic acid + 0.129 UDP + 0.129 ADP + 0.129 Orthophosphate + 6.216 CMP + 6.216 Diphosphate

**References**

Borodina I, Krabben P, Nielsen J (2005) Genome-scale analysis of Streptomyces coelicolor A3(2) metabolism. Genome Research 15 (6):820-829

Ingraham JL, Maaloe O, Neidhart FC (1983) Growth of the bacterial cell. Sinauer Associates, Inc, Massachusetts
